# Anatomic change over the course of treatment for non–small cell lung cancer patients and its impact on intensity-modulated radiation therapy and passive-scattering proton therapy deliveries

**DOI:** 10.1186/s13014-020-01503-9

**Published:** 2020-03-05

**Authors:** Mei Chen, Jinzhong Yang, Zhongxing Liao, Jiayi Chen, Cheng Xu, Xiaodong He, Xiaodong Zhang, Ronald X. Zhu, Heng Li

**Affiliations:** 1 Department of Radiation Oncology, Ruijin Hospital, Shanghai Jiao Tong University School of Medicine, Shanghai, 200025 China; 2 Department of Radiation Physics, The University of Texas MD Anderson Cancer Center, Houston, TX 77030 USA; 3 Department of Radiation Oncology, The University of Texas MD Anderson Cancer Center, Houston, TX 77030 USA; 4 Department of Radiation Oncology and Molecular Radiation Sciences, Johns Hopkins University School of Medicine, Baltimore, MD 21205 USA

**Keywords:** Anatomic change, Proton therapy, Adaptive therapy

## Abstract

**Purpose:**

To quantify tumor anatomic change of non-small cell lung cancer (NSCLC) patients given passive-scattering proton therapy (PSPT) and intensity-modulated radiation therapy (IMRT) through 6–7 weeks of treatment, and analyze the correlation between anatomic change and the need to adopt adaptive radiotherapy (ART).

**Materials and methods:**

Weekly 4D CT sets of 32 patients (8/8 IMRT with/without ART, 8/8 PSPT with/without ART) acquired during treatment, were registered to the planning CT using an in-house developed deformable registration algorithm. The anatomic change was quantified as the mean variation of the region of interest (ROI) relative to the planning CT by averaging the magnitude of deformation vectors of all voxels within the ROI contour. Mean variations of GTV and CTV were compared between subgroups classified by ART status and treatment modality using the independent *t*-test. Logistic regression analysis was performed to clarify the effect of anatomic change on the probability of ART adoption.

**Results:**

There was no significant difference (*p* = 0.679) for the time-averaged mean CTV variations from the planning CT between IMRT (7.61 ± 2.80 mm) and PSPT (7.21 ± 2.67 mm) patients. However, a significant difference (*p* = 0.001) was observed between ART (8.93 ± 2.19 mm) and non-ART (5.90 ± 2.33 mm) patients, when treatment modality was not considered. Mean CTV variation from the planning CT in all patients increases significantly (*p* < 0.001), with a changing rate of 1.77 mm per week. Findings for the GTV change was similar. The logistic regression model correctly predicted 71.9% of cases in ART adoption. The correlation is stronger in the PSPT group with a pseudo R^2^ value of 0.782, compared to that in the IMRT group (pseudo R^2^ = 0.182).

**Conclusion:**

The magnitude of target volume variation over time could be greater than the usual treatment margin. Mean target volume variation from the planning position can be used to identify lung cancer patients that may need ART.

## Introduction

External beam radiation therapy is an important technique in the management of lung cancer. Advanced beam delivery approaches such as intensity-modulated radiation therapy (IMRT) and proton therapy could improve dose conformality by creating a sharper dose gradient, which could, in turn, lead to reduced normal-tissue dose and facilitating safe dose escalation within the tumor [1, 2]. However, a sharper dose gradient demands a more precise dose delivery. Geometric uncertainties, especially intra-fractional tumor motion and inter-fractional anatomic changes, pose barriers to the accuracy of highly conformal treatment [3, 4]. Reduction of geometric uncertainties is therefore imperative to improve the therapeutic ratio, and studies have suggested that the combination of advanced image guidance and motion management can improve local control and overall survival for patients with non–small cell lung cancer (NSCLC) [5, 6].

Major concerns remain for treating NSCLC patients using proton therapy due to the sensitivity of proton dose deposition toward any beam path-length change, which could be induced by intra- and inter-fractional motion for NSCLC patients [4]. The effect of intra-fractional tumor motion can be mitigated using pre-treatment four-dimensional (4D) computed tomography (CT) imaging and 4D CT–based treatment planning. Respiration-correlated 4D CT shows the full range of possible positions of the tumor and critical organs, making it feasible to incorporate motion uncertainties into contouring, treatment planning, and treatment plan evaluation [7–9].

Adaptive radiotherapy (ART), which modifies the treatment plan to accommodate inter-fractional variation, is often used to mitigate uncertainties caused by anatomic changes. Dose benefits and better clinical outcomes were observed in lung cancer patients receiving ART than in those in a non-ART group [10, 11]. ART for lung cancer was reported to reduce mean lung dose, volume of lung receiving 20 Gy (V20Gy), mean esophageal and heart dose, and maximum spinal cord dose [12, 13]. Compared with a non-ART group, the implementation of ART strategies including soft-tissue tumor matching for patients with locally advanced lung cancer increased the rate of local-regional control without increasing treatment-related toxicity [14]. The typical implementation of ART generally includes repeated CT simulation, re-delineation of structures, plan re-optimization, and successive plan review and approval, which increase the workload of therapists, dosimetrists, physicists, and physicians.

Schmidt *et. al.* compared the dosimetric impacts of respiratory motion, inter-fractional baseline shifts, and anatomic changes in NSCLC patients treated with IMRT using daily cone beam CT (CBCT) and mid-treatment 4D CT scans and found that anatomic changes had more impact on patient dose than did internal target motion. They also suggested that ART can be used to achieve better target coverage throughout the treatment course [15]. Therefore, it is important to identify patients who need ART and predict the optimal time in the course of treatment for implementing ART. While previous studies have focused on addressing the requirement for ART with regard to anatomic changes, predictors of the need for ART planning remain unclear, and ART adoption largely depends on physicians’ discretion.

ART is used more often in proton therapy than in IMRT: ~ 30% of lung cancer patients who received proton therapy required ART, as opposed to ~ 10% of those who received IMRT [16, 17]. The greater need for ART in proton therapy could be due to the proton beams’ depth dose characteristics and greater sensitivity to path length change compared with photon beams. However, the imaging data for proton radiotherapy patients is very limited compared to imaging data for photon radiotherapy patients, and there is a lack of quantitative studies comparing sensitivity towards anatomic change between proton and photon radiotherapy. The main purpose of the current study is to understand if there were any fundamental difference in terms of imaging response between proton and photon radiotherapy, and if patient imaging from photon radiation could be used for retrospective proton ART planning studies. To that end, we hypothesize that by delivering the same dose fractionation to the same target volume, there will be no observable imaging response difference using the two delivery techniques over a certain period of time during the treatment, with or without ART.

In addition, new planning techniques such as robust optimization and multiple CT optimization require a quantitative understanding of anatomic change so that these factors can be explicitly accounted for during planning [17–19]. If anatomic variations could be identified before treatment starts, or if there were a better understanding of the magnitude of anatomic change in the patient over time, then the initial treatment plan could be made more robust against subsequent anatomic change. However, accounting for the anticipated anatomic change in treatment planning is currently not feasible in clinical practice because information about the anatomic change embedded in the adaptive CT cannot be obtained until the patient is under treatment.

The current study aimed to bridge these knowledge gaps and identify predictors of the need for ART by quantifying the anatomic variations of patients treated with IMRT and passive-scattering proton therapy (PSPT) over the course of treatment and analyzing the correlation of these variations with ART adoption.

## Materials and methods

### Patient selection

Repeated 4D CT sets were retrospectively reviewed for 32 lung cancer patients enrolled in a prospective randomized trial (NCT00915005), that compared the outcomes of PSPT and IMRT for inoperable NSCLC. All patients received radiotherapy at our institution during 2009 through 2014. Details of the trial have been recently published [18, 20]. Of the 212 NSCLC patients enrolled in the trial, 136 received IMRT and 76 received PSPT. Each patient received weekly CT or CBCT (only available for IMRT patients) during their treatment. If the weekly scans showed a change in iGTV coverage (defined as < 95% target volume receiving 95% of the prescribed dose, or more than 2 cm^3^ receiving more than 120% of the prescribed dose), then ART was deemed necessary. Sixteen patients (12%) who received IMRT and 22 patients (29%) who received PSPT required at least one adaptive planning based on the weekly images. The rate of adaptive planning is consistent with other NSCLC patients treated at our center. Eight patients in the IMRT group and eight patients in the PSPT group required ART during their treatment were selected in this retrospective study. A same number of patients in the IMRT and PSPT arms, who did not need adaptive therapy, with GTV volume matched with the patients who needed adaptive therapy were also chosen for comparison. For each patient, the tumor was prescribed a total dose of 60–74 Gy (RBE) in 30–37 fractions over 6–7 weeks at 2 Gy (RBE) per fraction. Gy (RBE) is used in proton therapy prescription; it is the product of physical dose (Gy) and relative biologic effectiveness (RBE) for protons.

### Image acquisition

Before treatment, all patients underwent 4D CT simulation for target delineation, motion assessment, and treatment planning. In addition to the pre-treatment planning 4D CT, each patient had 2–7 weekly 4D CTs. The weekly CT was rigidly registered to the planning CT in a commercial treatment planning system (TPS, Pinnacle^3^, Philips Healthcare, Andover, MA, for IMRT planning; Eclipse, Varian Medical Systems, for PSPT planning), and the original contours on the planning CT were then deformed to the weekly CT using an in house deformable image registration software [21]. A verification plan was generated by copying the treatment plan’s beam data to the newly acquired CT, and the dose distribution was recalculated without optimization. If violation of dose constraints in target and/or normal tissues was observed, physicians requested ART on the weekly CT with new contours.

The 4D CT data sets were obtained in cine mode on a multislice CT scanner (Discovery ST or Lightspeed RT16, GE Healthcare, Chicago, IL). During each 4D CT scan, 10 three-dimensional (3D) CT sets reflecting 10 equally spaced phases during the breathing cycle were generated, along with maximum intensity projection (MIP) and average intensity projection (AIP) images. All images were uploaded to the PACS (picture archive and communication system) and then exported to the TPS.

### Target definition

All the 3D CT sets reconstructed in one 4D CT scan were inherently registered to each other. The gross tumor volume (GTV) was initially delineated on MIP, T0 (end-inspiration, GTV_T0) and T50 (end-expiration, GTV_T50) phases; combined as internal gross tumor volume (IGTV); validated on all other phases; and then transferred to the AIP images for treatment planning. In the treatment plan of PSPT, the CT voxels inside the IGTV was overridden with a constant density to account for the intra-fractional motion effect [7]. The clinical target volume (CTV) was created by isotropically expanding the GTV by 8 mm. Mean variations of the CTV on the T50 phase (CTV_T50, created by expanding GTV_T50) were used for analyzing the deformation changes of the target during treatment.

### Image registration

Images of the T50 phase of the weekly CT (moving CT) were registered to the planning CT (reference CT) using a rigid/deformable registration algorithm developed in house with MATLAB (Mathworks, Inc., Natick, MA). After the format consistency between the reference CT and moving CT was checked, rigid registration was performed to determine the isocenter shift between the reference CT and moving CT. The isocenter shift was then used to perform 3D Demons-based deformable registration [21], generating a deformation vector field between the reference CT and the moving CT.

### Quantification of anatomic change

The variation between CTs for any given region of interest (ROI) was determined using all voxels of the deformation vector field within the ROI contour. For example, the weekly mean variations of the CTV relative to the initial CT were quantified as the mean value of the magnitude of the deformation vectors for all voxels within the CTV contours. Each vector magnitude is the square root of the sum of the squared *x* (lateral), *y* (anterior-posterior), *z* (superior-inferior) displacement values. To evaluate the deformation trend between treatment modalities and ART status, weekly magnitudes averaged over the patients in each group were calculated. The rigid and deformable registration between CT data sets and data extraction for all patients were completed automatically via batch processing MATLAB code developed in house.

### Data analysis

Differences in the mean variations of the CTV during the treatment were analyzed using a linear mixed-effects regression model that estimated patient variation as a random effect. Independent *t*-tests were performed to compare the mean CTV variations between

1. patients with and without ART plans in the entire cohort,
2. patients with and without ART plans in the IMRT group,
3. patients with and without ART plans in the PSPT group,
4. patients given IMRT and those given PSPT in the ART group, and
5. patients given IMRT and those given PSPT in the non-ART group.

The statistical significance level was set at 0.05. All statistical analysis was done with SPSS version 24.0 (IBM, Chicago, IL). We performed comparisons 1, 2, and 3 to determine whether the mean CTV variation from planning CT was a good indicator of ART strategy. In addition, logistic regression was performed to ascertain the effect of tumor positional variations on the likelihood that patients use ART. By performing comparisons 4 and 5, we investigated the difference between two treatment technologies with regard to the tolerance of patient anatomical change as represented by mean CTV variations from the planning CT.

## Results

### Patient data

The clinical characteristics of the patients are summarized in Table 1.

**Table 1:** Patients’ clinical characteristics

Characteristic	All (*n* = 32)	PSPT (*n* = 16)	IMRT (*n* = 16)
ART status, no.			
With ART	16	8	8
No ART	16	8	8
4DCT sets per patient, median (range)	7 (2–7)	7 (7–7)	6.5 (2–7)
Sex, no.			
Male	19	11	8
Female	13	5	8
Age, median (range), years	64 (43–78)	64 (43–76)	62 (47–78)
Disease stage, no.			
IV	5	1	4
IIIB	21	12	9
IIIA	5	2	3
IIA	1	1	0
Target volume, median (range), cm^3^			
GTV	221.9 (12.2–686.6)	127.1 (22.7–673.7)	280.9 (12.2–686.6)
CTV	446.3 (120.9–1329.3)	398.7 (131.7–1329.3)	558.2 (120.9–1245.7)

*IMRT* intensity-modulated (photon) radiation therapy, *PSPT* passive-scattering proton therapy, *GTV* gross tumor volume, *CTV* clinical target volume, *ART* adaptive radiotherapy

### Accuracy of deformable image registration

**Fig. 1:**
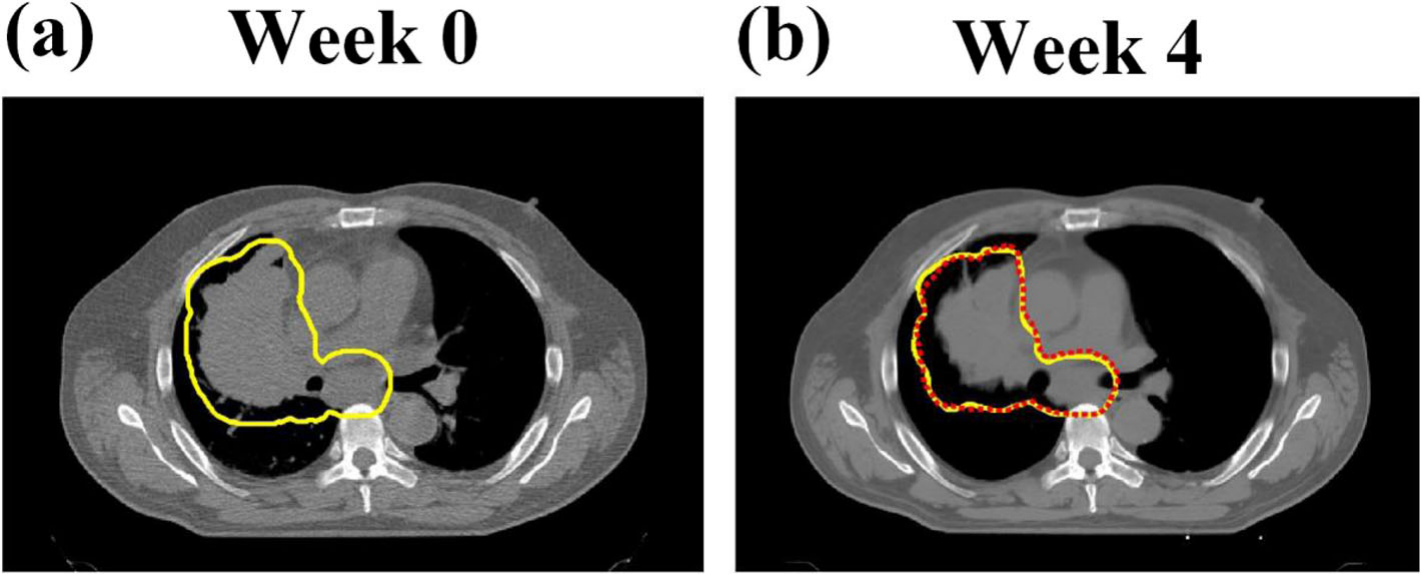
Clinical target volume (CTV) contour on the CT acquired at weeks 0 (planning CT) **a** for a typical patient enrolled in the passive scattering proton therapy (PSPT) group, who received adaptive radiotherapy (ART) at week 4. The deformed contour (red dash line) was compared with the clinical approved contour on the CT acquired at week 4 **b**

The Demons-based deformable registration has been validated in multiple scenarios with reasonably good results [22–26]. Here, we illustrated the comparison of deformed contour and clinical approved contour for a typical PSPT patient who underwent ART at week 4 in Fig. 1. The Dice similarity coefficient between the two contours shown in Fig. 1b is 93.68%.

### Mean target variations from planning CT

**Fig. 2:**
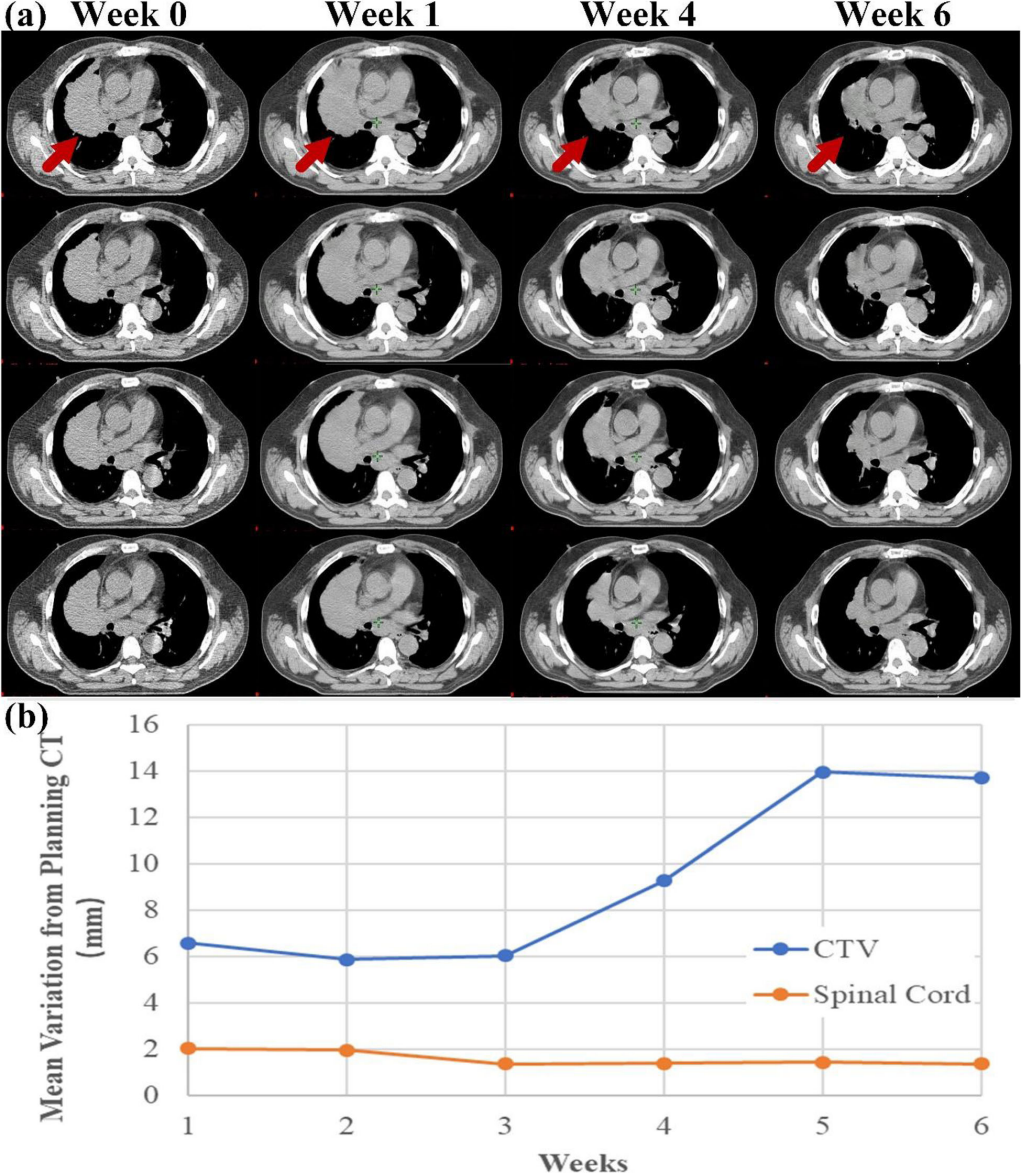
Anatomic change and mean variations in a typical patient enrolled in the passive scattering proton therapy (PSPT) group, who received adaptive radiotherapy (ART) at week 4. **a** Anatomic changes in transverse planes of the representative CT slides from the T50 (end-expiration) series acquired at weeks 0 (planning CT), 1, 4, and 6. **b** Mean variations in the clinical target volume (CTV) and spinal cord from planning CT over the course of treatment

Figure 2 shows registration and analysis results for the same patient illustrated in Fig. 1. These results include anatomic changes over the course of 6 weeks of treatment (Fig. 2 a, as well as variations in the CTV, which changed drastically after week 4, and in the spinal cord, which remained stable throughout the treatment (Fig. 2 b.

The mean GTV and CTV variations from the planning CT over time are shown in Table 2.

**Table 2:** Mean CTV variations from the planning CT over time for the entire cohort

	Weeks (deviations in mm)	1 (mm)	2 (mm)	3 (mm)	4 (mm)	5 (mm)	6 (mm)
GTV	Entire cohort	4.85 ± 2.03	6.61 ± 3.01	7.06 ± 3.23	7.92 ± 3.78	8.19 ± 4.18	8.98 ± 4.55
	With ART	5.98 ± 2.17	7.58 ± 2.82	9.04 ± 3.23	9.91 ± 3.04	10.31 ± 3.47	11.46 ± 4.54
	No ART	3.86 ± 1.28	5.65 ± 3.01	5.20 ± 1.88	6.06 ± 3.50	6.34 ± 3.92	6.81 ± 3.38
CTV	Entire cohort	4.90 ± 1.90	6.39 ± 2.82	6.87 ± 3.06	7.51 ± 3.48	7.79 ± 3.88	8.50 ± 4.18
	With ART	5.99 ± 1.97	7.22 ± 2.62	8.56 ± 3.27	9.32 ± 2.81	9.75 ± 3.37	10.71 ± 4.45
	No ART	3.94 ± 1.24	5.66 ± 2.87	5.28 ± 1.79	5.81 ± 3.24	6.08 ± 3.54	6.56 ± 2.84

*GTV* internal gross tumor volume, *CTV* clinical target volume, *ART* adaptive radiotherapy

### Time trend of the mean target variations

**Fig. 3:**
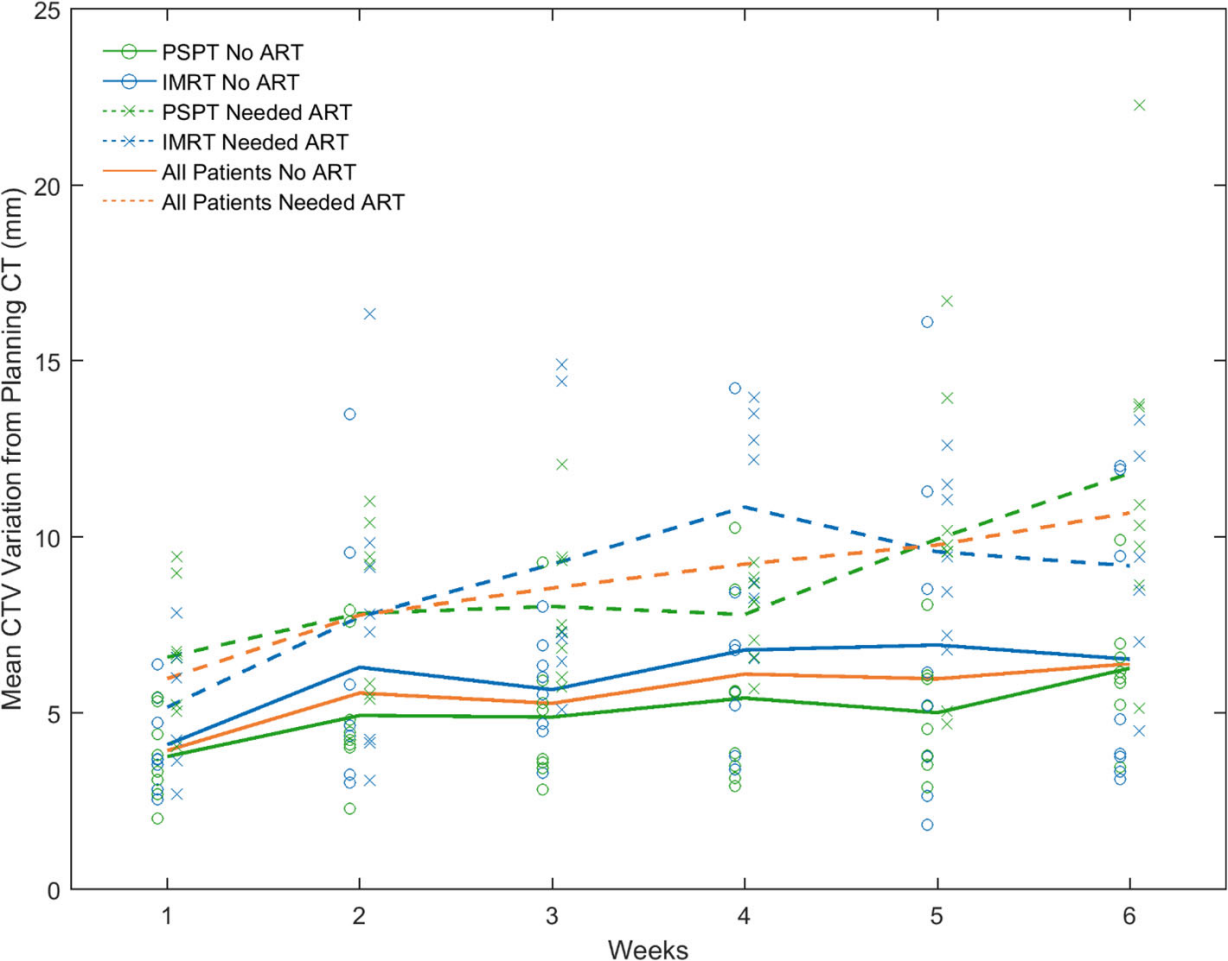
Mean CTV variations each week for the entire cohort, the PSPT group, and the intensity-modulated radiation therapy (IMRT) group

As treatment proceeded, both mean GTV and mean CTV variation from the planning CT increased significantly (*p* < 0.001), at a rate of 1.86 and 1.76 mm per week, respectively. The mean (±standard deviation) initial variations (in week 1) for GTV and CTV in all patients were 4.85 ± 2.03 mm (range 1.85–10.27 mm) and 4.90 ± 1.87 mm (range 2.01–9.44 mm), respectively. The mean GTV and CTV variations in week 6 were 8.98 ± 4.55 mm (range 3.23–21.35 mm) and 8.50 ± 4.18 mm (range 3.12–22.26 mm), respectively. The time trend of the GTV and CTV variations did not differ significantly (*p* = 0.773), nor was there a significant difference between the time-averaged variations of the two ROIs (*p* = 0.844). The weekly mean variation in CTV from the planning CT for all patient groups is shown in Fig. 3.

In both the ART and non-ART groups, the mean CTV variations significantly increased over weeks. The rate of increase in the ART group was 2.16 mm per week (*p* < 0.001) and that in the non-ART group was 1.36 mm per week (*p* < 0.001). However, the rate of increase between the two groups is non-significantly different (*p* = 0.691).

### Time-averaged mean target variations

Table 3 shows the comparison of time-averaged mean GTV and CTV variations from the planning CT between the ART and non-ART groups in the whole cohort and in modality-stratified subgroups.

**Table 3:** Time-averaged mean GTV and CTV variations from the planning CT within different groups

	Group	Non-adaptive (mm)	Adaptive (mm)	*p* value
GTV	Entire cohort	6.07 ± 2.63	9.41 ± 2.17	0.001
	PSPT	5.20 ± 1.66	9.76 ± 2.01	< 0.001
	IMRT	6.94 ± 3.21	9.06 ± 2.41	0.159
CTV	Entire cohort	5.90 ± 2.33	8.93 ± 2.19	0.001
	PSPT	5.19 ± 1.59	9.24 ± 1.85	< 0.001
	IMRT	6.61 ± 2.82	8.63 ± 2.57	0.156

*GTV* internal gross tumor volume, *CTV* clinical target volume, *IMRT* intensity-modulated (photon) radiation therapy, *PSPT* passive-scattering proton therapy

Again, the results for GTV and CTV are similar. Overall, patients with ART plans showed a significantly larger displacement, at 9.41 ± 2.17 mm for GTV and 8.93 ± 2.19 mm for CTV, respectively, than did patients without ART plans, whose displacement was 6.07 ± 2.62 mm for GTV and 5.90 ± 2.33 mm for CTV (*p* = 0.001), respectively, without taking treatment modality into consideration. The minimum CTV shifts were 3.43 mm in the ART group and 6.47 mm in the non-ART group. Similarly, in the PSPT group, CTV variation from planning CT significantly differed between patients with and without ART plans (*p* < 0.001). However, in the IMRT group, in which mean CTV variations from the planning CT were 6.61 ± 2.82 mm in the ART group and 8.63 ± 2.57 mm in the non-ART group, the difference was non-significant (*p* = 0.156). Nor did the IMRT and PSPT subgroups significantly differ with regard to CTV variation from the planning CT in either the ART (*p* = 0.596) or non-ART (*p* = 0.241) cohort.

The time-averaged mean CTV variation from the planning CT was 7.41 ± 2.71 mm among all patients. These variations did not significantly differ between patients given IMRT (7.61 ± 2.80 mm) and those given PSPT (7.21 ± 2.67 mm; *p* = 0.679), regardless of ART status, indicating that the magnitude of patient anatomic change over the course of treatment was similar across treatment modalities.

### Correlation of mean CTV variation with ART adoption

**Fig. 4:**
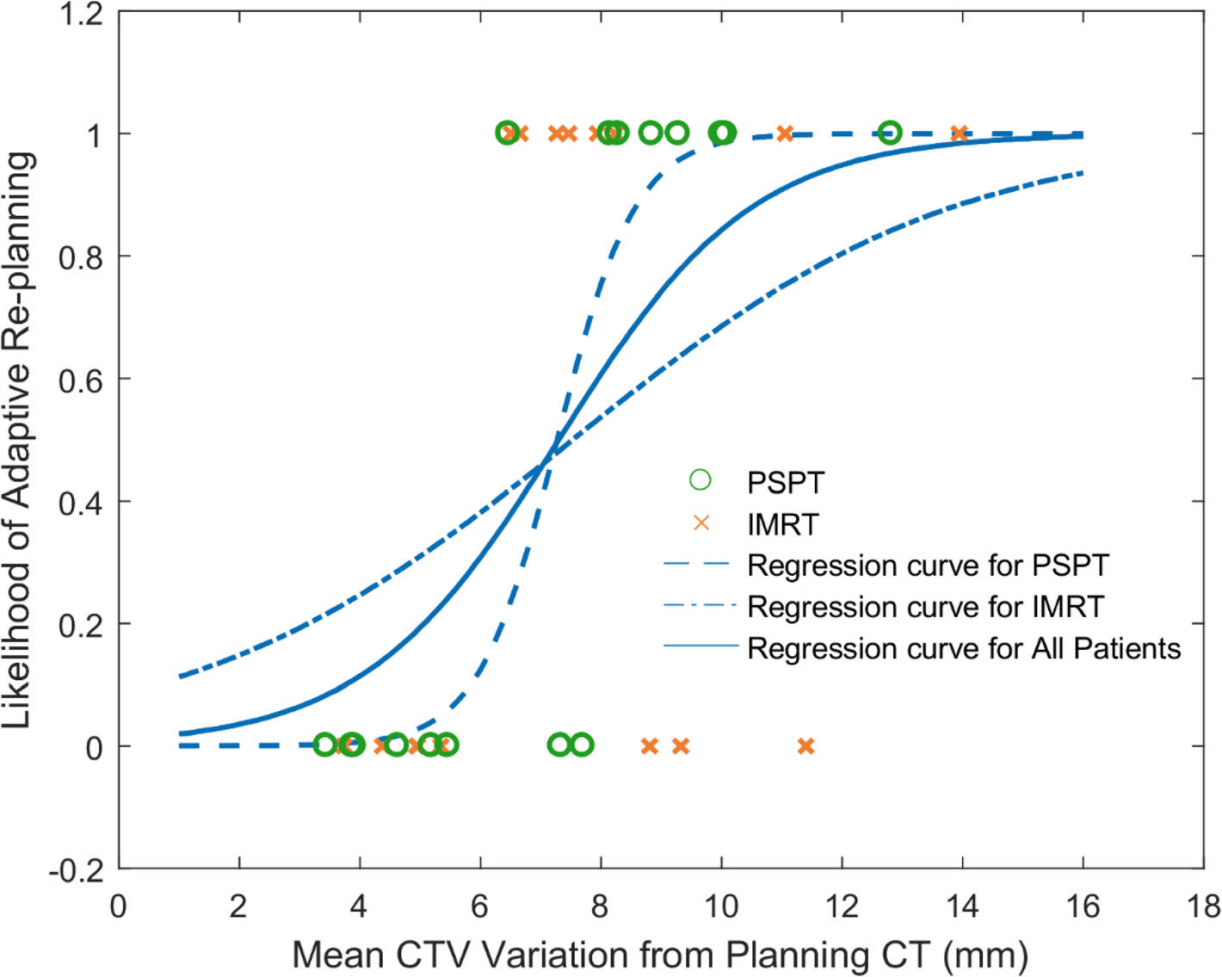
Likelihood of adaptive re-planning versus time-averaged mean CTV variance from planning CT. The S curves are the logical regression functions fitted to the binary data for all patients, the PSPT group, and the IMRT group

Figure 4 shows the logistic regression curves for the entire cohort, the PSPT group, and the IMRT group.

The figure shows a statistically significant relationship between an increasing mean CTV variance from planning CT and an increasing likelihood of ART treatment for the entire cohort (*p* = 0.006). The model correctly predicted 71.9% of cases and explained 42.8% of the variance in ART strategy adoption. The correlation was stronger in the PSPT group (pseudo R^2^ = 0.782) than in the IMRT group (pseudo R^2^ = 0.182). A higher incidence of ART planning was observed in week 4, accounting for more than half of the cases.

## Discussion

In this quantitative study of relationship between ART and magnitude of inter-fractional anatomic variation over the course of treatment, the time-averaged mean CTV variations from the planning CT did not significantly differ between the IMRT (7.61 ± 2.80 mm) and PSPT (7.21 ± 2.67 mm) groups, but did significantly differ between the ART (8.93 ± 2.19 mm) and non-ART (5.90 ± 2.33 mm) groups. These results indicate that patient anatomy changes substantially over the course of treatment regardless of treatment modality and provide a quantitative foundation for improving treatment planning techniques. Our results also suggests that it is indeed feasible to use imaging data from photon radiotherapy patients, which is more readily available, for retrospective proton ART planning studies, as there is no significant difference was identified between the imaging responses of the target in patients treated the two different modalities.

We also investigated the influence of anatomic change on the need for ART in the context of treatment modality. Among the patients given IMRT, mean CTV variations from the planning CT did not significantly differ between the ART and non-ART groups, suggesting that IMRT is more robust to anatomic changes than is PSPT.

It is essential to adapt treatment planning to tumor motion and anatomic change in radiation treatment for lung cancer. Unlike respiratory motion, which can be incorporated in the initial treatment plan via 4D CT, anatomic change can currently be compensated for only by adaptive re-planning on a new CT. Moreover, compared with photon therapy, proton radiotherapy is more sensitive to density variation in the beam path. In the presence of setup uncertainties and anatomic changes, a higher frequency than photon therapy of re-imaging and re-planning has been recommended in proton therapy [16]. There are ~ 30% of lung cancer patients who received proton therapy required ART, as opposed to ~ 10% of those who received photon therapy [16, 17, 27]. However, the optimal timing of ART for each treatment technique with regard to anatomic change is unknown. In addition, the initial treatment plan with robust optimization may not be able to account for anatomic variations induced by treatment [17]. To ensure tumor coverage and normal tissue sparing, patients must undergo periodic CT imaging, and clinical staff, including a dosimetrist, a physicist, and a physician, must work on several verification plans on the repeated CT set regardless of treatment technique.

In previous studies, anatomic change was commonly quantified by tumor volume on repeated CT images, which could be performed using fluorodeoxyglucose positron emission tomography (FDG PET)/CT, CBCT, megavoltage CT, or 4D CT. The target was re-contoured on every periodic image set by physicians. The additional delineation was not only time-consuming but also found to be associated with additional uncertainty [28]. We demonstrated the correlation of ART strategy use with anatomic change in the tumor, characterized as the mean CTV deviation on weekly 4D CT imaging. CTV displacement was calculated by averaging the magnitude of the deformation vector rather than relying on target contours. Both the magnitude of anatomic change and the rate of change over time for CTV volume were much greater than those of a more stable volume, e.g., spinal cord (Fig. 2b). While the CTV change for patients in the non-ART group (5.90 ± 2.33 mm) was close to our usual planning margin with image guidance (planning target volume or CTV to PTV expansion, and setup uncertainty of 5 mm for IMRT and PSPT, respectively), the CTV change in the adaptive group was clearly more than the established planning margins; therefore, new planning techniques are needed to account for these changes.

Mixed-effects linear regression showed that mean CTV variation from the planning CT increased significantly over the course of treatment, at 1.77 mm per week. The rate of increase in the ART group was higher than that in the non-ART group. Similar time trends were seen in patients given IMRT and those given PSPT. These results are similar to the findings of Britton et al. [29], who observed a significant (*p* = 0.049) increase over the course of treatment in GTV and internal target volume centroid variation in a retrospective study of eight patients with non–small cell lung cancer, in which weekly CT was rigidly registered to planning CT and the position of the target centroid was measured on every image set. Hassbeek et al. [30] and Sun et al. [31], using a similar method but confined to a short treatment course of hyperfractional radiotherapy, reported a limited time trend in the mean 3D displacement of the PTV and GTV centroid. However, these previous studies captured only the tumor contour changes over time and disregarded tumor density variation and changes outside of the target, which could both be relevant to radiotherapy, especially proton therapy. In the current study, we used deformable registration to acquire patient anatomic change over time for each voxel and thus were able to capture tumor deformation information, such as tumor shrinkage, growth, and displacement, that may not be feasible to measure using centroid position changes only.

The action level of ART adoption varies with institutional practice and treatment delivery technique. Van den Bosch et al. [32] developed an automatic method to select patients eligible for ART with an accuracy of 79%. The criterion set was the change in the water-equivalent path length to the edge of the target on daily CBCT. In the present, single-center study, mean CTV variation from the planning CT was found to significantly differ between ART and non-ART groups on an independent *t*-test, which indicates that mean CTV variation from the planning CT could be a good predictor of the need for ART. The correlation coefficient between CTV variation from the planning CT and ART treatment was 0.43, and the predictive accuracy of the logistic regression model was 71.9%. Plan updates may be warranted when the 3D shift of mean CTV variation from the planning CT exceeds 8.26 mm, the median such variance for the ART group. Studies by Berkovic et al. [33] and Britton et al. [29] suggested that the optimal time point for ART was around fraction 15, after week 3. However, in our study, up to 60% of the ART re-planning occurred in week 4. ART decision-making is affected by multiple parameters, including patient weight change, pleural effusion, atelectasis, dose-volume histogram, dose distribution, potential clinical gain, and availability of clinical resources. Additional evidence is required to back any single indicator for formulating a patient-specific ART strategy. Correlations between dose change, treatment outcome, and anatomic variation need to be investigated in further studies.

Due to the purpose of the study, patient selection was difficult due to the limited patient size in the ART arm, and the different percentage of patients required ART between IMRT and PSPT modalities. Since the ART status was unknown when the patient was randomized to receive IMRT or PSPT, the anatomy change should not be dependent on treatment modality, which was confirmed by our results. We acknowledged that the effectiveness of current approach is limited by the registration error of the deformable image registration. The Demons registration algorithm used in this study has been fully validated for 4D CT registration. In our previous study, the mean registration accuracy of 300 landmark pairs was within 1.3 mm on ten 4D CT datasets from deformable image registration lab (http://dir-lab.com) [34]. This algorithm has also achieved registration accuracy of within 1.1 mm when comparing the calculated deformation and the known deformation in the voxel-level validation [24]. The performance of deformable image registration varied with registration scenarios and may not work properly in the presence of unexpected anatomy changes. For all registrations in the work, we randomly spot checked one registration for each patient using the overlapped contours to ensure no gross registration errors. Also, it worth mentioning that, deformable registration is an ill-posed problem with multiple solutions. The registration results rely on the underlying assumption of the regularization on the registration. The voxel-level deformation vectors might not reflect the actual anatomy change at the voxel location due to uncertainties; however, using the mean of deformation vectors inside an ROI could minimize the impact from uncertainties, thus approximating the actual anatomy changes of the ROI, as what we did in this project. Another limitation of the current study is that only inter-fractional changes in the patient were investigated. Although previous studies showed intra-fractional respiratory motion produces only relatively minor variations, it could still bring about dosimetric changes, which is the main clinical criterion for ART decision-making. Refraining from considering intra-fractional variations would slightly weaken the ability of inter-fractional changes to predict ART need. A more straightforward and effective method for identifying patients needing ART would be to explore the quantitative dose variation of the target and normal tissues in a large cohort on the basis of the deformable registration methods presented in this study.

## Conclusion

The magnitude of CTV variation over time could be greater than the usual treatment margin. Mean CTV deviation from the planning position can be used to identify lung cancer patients who need ART. This indicator has stronger predictive power for ART decision-making in patients receiving PSPT than in those receiving IMRT. Further research into image and dose prediction is needed for the efficient implementation of ART.

## Data Availability

The datasets related to patient information are not available. Other data used and/or analyzed during the current study are available from the corresponding author on reasonable request.

## Abbreviations

4D CT: Four-dimensional computed tomography
AIP: Average intensity projection
ART: Adaptive radiotherapy
CBCT: Cone beam computed tomography
CTV: Clinical target volume
GTV: Gross tumor volume
IGTV: Internal gross tumor volume
IMRT: Intensity-modulated radiation therapy
MIP: Maximum intensity projection
NSCLC: Non-small cell lung cancer
PSPT: Passive-scattering proton therapy
RBE: Relative biologic effectiveness
ROI: Region of interest
